# New Symmetrically Esterified m-Bromobenzyl Non-Aminobisphosphonates Inhibited Breast Cancer Growth and Metastases

**DOI:** 10.1371/journal.pone.0004685

**Published:** 2009-03-05

**Authors:** Mohamed Abdelkarim, Erwann Guenin, Odile Sainte-Catherine, Nadejda Vintonenko, Nicole Peyri, Gerard Yves Perret, Michel Crepin, Abdel-Majid Khatib, Marc Lecouvey, Mélanie Di Benedetto

**Affiliations:** 1 Université Paris 13, CNRS FRE CSPBAT, Laboratoire de Chimie Structurale Biomoléculaire, Laboratoire de pharmacologie, Université Paris 13, UFR SMBH, Bobigny, France; 2 INSERM 553 Endothélium et Angiogénèse Laboratoire d'Hémostase, Paris, France; 3 INSERM/UP7 UMRS 940. Equipe Avenir, IGM, Paris, France; University of Hong Kong, Hong Kong

## Abstract

**Background:**

Although there was growing evidence in the potential use of Bisphosphonates (BPs) in cancer therapy, their strong osseous affinities that contrast their poor soft tissue uptake limited their use. Here, we developed a new strategy to overcome BPs hydrophilicity by masking the phosphonic acid through organic protecting groups and introducing hydrophobic functions in the side chain.

**Methodology/Principal Findings:**

We synthesized non-nitrogen BPs (non N-BPs) containing bromobenzyl group (BP7033Br) in their side chain that were symmetrically esterified with hydrophobic 4-methoxphenyl (BP7033BrALK) and assessed their effects on breast cancer estrogen-responsive cells (T47D, MCF-7) as well as on non responsive ones (SKBR3, MDA-MB-231 and its highly metastatic derived D3H2LN subclone). BP7033Br ALK was more efficient in inhibiting tumor cell proliferation, migration and survival when compared to BP7033Br. Although both compounds inhibited tumor growth without side effects, only BP7033Br ALK abrogated tumor angiogenesis and D3H2LN cells-induced metastases formation.

**Conclusion/Significance:**

Taken together these data suggest the potential therapeutic use of this new class of esterified Bisphosphonates (BPs) in the treatment of tumor progression and metastasis without toxic adverse effects.

## Introduction

Bisphosphonates (BPs) had long been used in metabolic bone disease as osteoporosis, tumor-associated hypercalcaemia and metastases-induced osteolysis due to their ability to inhibit bone resorption. BPs were able to bind divalent cations like Ca^2+^ or zinc constituting the basis of their bone-targeting property and their inhibition of the proteolytic activity of matrix metalloproteinases (MMP), respectively. The nature of their side chains gave rise to a variety of possible structures and stereochemistry determining their different potencies [1]–[4]. Non-nitrogen containing BPs (non N-BPs) acted by forming non hydrolysable ATP-analogues and were less effective than nitrogen-containing BPs (N-BPs) in inhibiting bone metastasis [5]. However, Zoledronate treatment of patients were reported to induce toxic side effect characterised by osteonecrosis of the jaw while non N-BP did not produce this effect [6], [7]. N-BPs, such as zoledronate, acted on the mevalonate pathway inhibiting the farnesyl diphosphate synthase (FPP) thereby depleting the cells of the farnesyl (FPP) or geranylgeranyl (GGPP) diphosphate isoprenoids [8]. Isoprenoids were required for translocation and anchorage of small G proteins like Rho or Ras to the plasma membrane assuring their ultimate involvement in signal transduction during several important normal and tumor cellular pathways.

**Figure 1:**
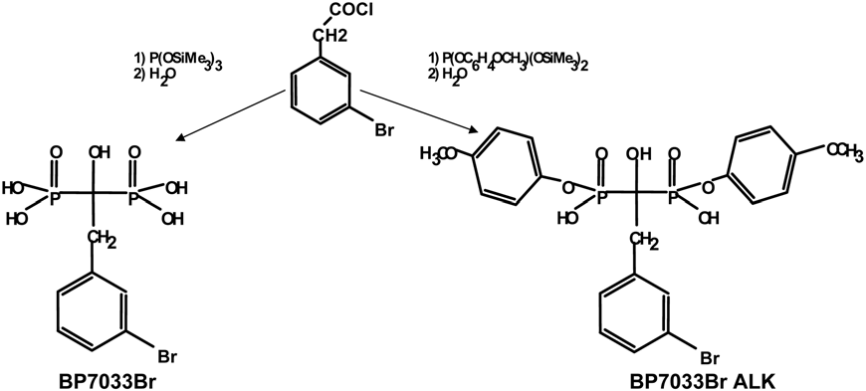
Chemical structure of BP7033Br and BP7033Br ALK. The first step (1) was an Arbusov reaction between an activated carboxylic function and a very reactive species, the bis(trimethylsilyl) phosphite and the second one (2) was hydrolysis.

However, *in vivo* efficacy of all BPs on extra-osseous sites or primary tumors was still debated. Only a small number of studies demonstrated their *in vivo* antiproliferative activity on tumors or metastasis present in soft tissues [9]. The reasons were the poor oral bioavaibility (0.3–7% in humans) due to chelation of metal ions by phosphonic acid group inside the digestive lumen, poor membrane permeability due to poor BP lipophilicity as well as strong uptake by bone tissue [10]. Previously, our laboratory developed a new strategy to overcome BP hydrophilicity by masking the phosphonic acid with organic protecting groups and introducing hydrophobic functions in the side chain [11]. We previously demonstrated that an esterified BP with methyl group displayed antitumor growth and antiangiogenic activities on A431 tumors being more effective *in vivo* than *in vitro* [12]. In order to further increase the lipophilicity of BPs (and their entering into the cells), we synthesized new aromatic 1-hydroxymethylene-1,1-bisphosphonic acids containing phenyl or halogen phenyl ring in their side chains. Interestingly, we showed that these compounds exhibited potent antiproliferative activities *in vitro* on human epidermoid A431 cells [13]. In parallel, recently crystallographic and computational investigation revealed that the presence of phenyl ring in the side chain permitted non N-BPs to interact with farnesyl enzyme [14]. Thus, we synthesized a class of BPs that contained bromobenzyl in their side chains (BP7033Br, Fig. 1). For the first time, we symmetrically esterified one of each phosphonic acids with aromatic groups (BP7033Br ALK, Fig. 1).

In this study we tested the effects of BP7033Br and BP7033Br ALK on MDA-MB-231 xenograft growth and metastasis. We found that the addition of hydrophobic bromobenzyl groups on non N-BPs side chain rendered them efficient in inhibiting estrogen responsive as well as non-responsive breast cancer cells like MDA-MB-231 and metastatic subpopulation (D3H2LN) cell growth as well as migration and invasion *in vitro*. Further, we demonstrated that BP7033Br and BP7033Br ALK differently reduced the proteolytic activity of MMP-9 and MMP-2. These two non-N BPs inhibited MDA-MB-231 xenografts associated with tumor angiogenesis reduction. Importantly, we demonstrated that only esterification of m-bromobenzyl bisphosphonate revealed to induce antimetastatic effect in nude mice.

## Results

### BP7033Br ALK was more potent than BP7033Br in inhibiting breast cancer cell viability

**Figure 2:**
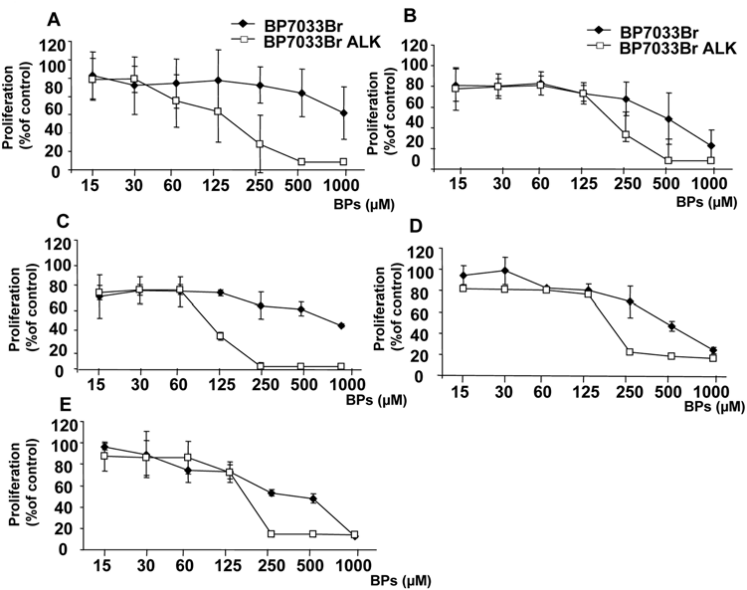
BP7033Br and BP7033Br ALK inhibited viability of different breast cancer cells. T47D (A), MCF-7 (B), SKBR3 (C), MDA-MB-231 (D) or D3H2LN cells (E) (1×10^5^) were treated with BP7033Br and BP7033Br ALK at increasing concentrations for 72 h. Then, the cells were washed and incubated with 0.1 mL of MTT (2 mg/mL) for 4 h. Optical density was measured at 570 nm using a Labsystems Multiskan MS microplate reader. Data represents the mean value (±SD) of three independent experiments.

We investigated the effect of BP7033Br and its esterified analogue BP7033Br ALK on the proliferation of several breast cancer cell lines with different estrogen-receptor statuses as T47D (Fig. 2A), MCF-7 (B), SKBR3 (C), MDA-MB-231(D) and its D3H2LN metastatic subclone (E). The special features of D3H2LN subclone are that these cells exhibited more important tumor growth than MDA-MB-231 and that when injected in the left heart ventricle, they induced faster metastasis and more multiple tissues sites [15]. After 72 h, BP7033Br differently inhibited the several cell lines. The maximal T47D cell viability inhibition induced by BP7033Br was about 38% and the IC_50_ was not reached even at the maximal concentration (1 mM). MCF-7 and SKBR3 cells were inhibited by 50% with 500 µM of BP7033Br and this concentration was the more effective for SKBR3 cells. Viability of both MDA-MB-231 and D3H2LN was inhibited by BP7033Br in a dose-dependent manner with the same efficacy (IC_50_ = 500 µM, Fig 2B). For these cells, the maximal effect (80%) was achieved at 1 mM of BP7033Br. Considering BP7033Br ALK, we showed that this esterified BP was more effective than BP7033Br on T47D, MCF-7 and SKBR3 cells with maximal effect achieved at 250 µM. BP7033Br ALK also inhibited the MDA-MB-231 and D3H2LN cell lines in a dose-dependent manner with the same efficacy (IC_50_ = 170 µM, Fig. 2D and E). In addition, BP7033Br ALK was at least 4-fold more efficient than non esterified BP7033Br since maximal inhibition of cell viability (90%) was reached at 250 µM. Breast cancer cell viability inhibitions induced by both BPs were also time-dependent and started after 24 h (data not shown).

### BP7033Br and BP7033Br ALK inhibited MDA-MB-231 breast cancer cell viability through different cell cycle arrests

**Figure 3:**
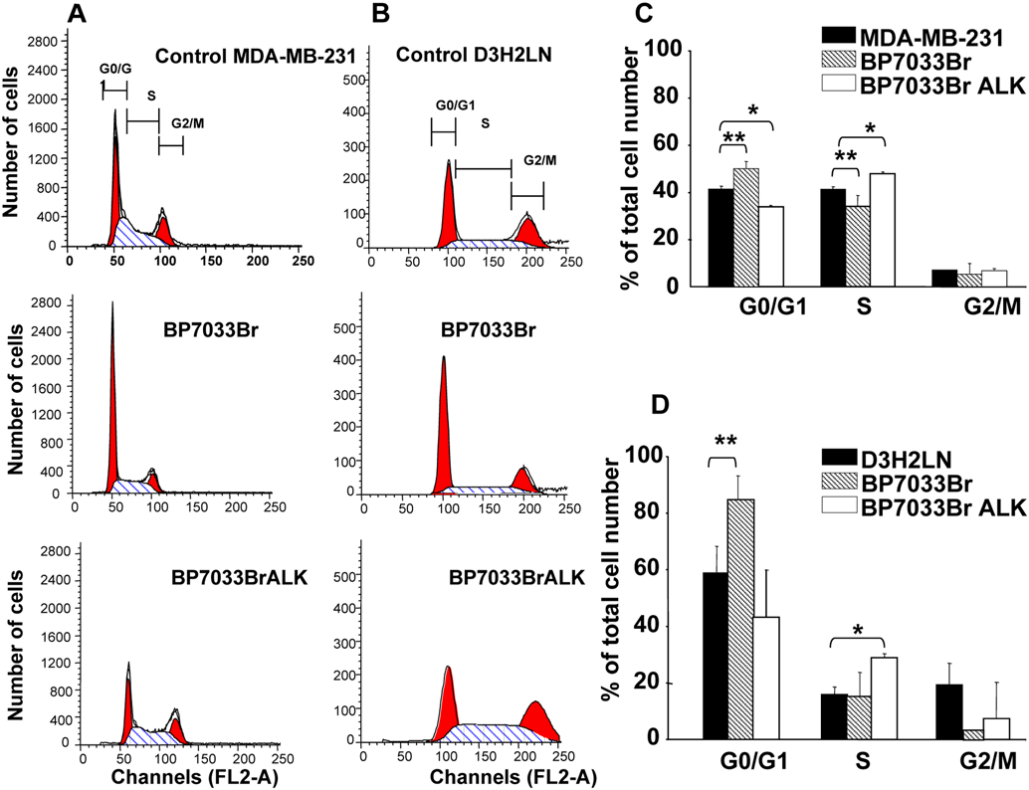
BP7033Br and BP7033Br ALK inhibited MDA-MB-231 breast cancer cell cycle progression. Distribution of MDA-MB-231 (A) and D3H2LN (B) cells treated with BPs in different cell cycle phases was determined as described in “Materials and Methods”. Histograms show the percentage of MDA-MB-231 (C) and D3H2LN (D) cell repartition. Each column represents a mean (±SD) of three independent experiments. *P _control versus BP7033BrALK_ and ** *P*_control versus BP7033Br_<0.05.

In order to understand the effect of the BPs on breast cancer cell viability, we evaluated their effect on cell cycle progression (Fig. 3). MDA-MB-231 and D3H2LN cells (Fig. 3A and 3B, respectively) were treated with effective doses of BP7033Br and BP7033Br ALK for 72 h (500 µM and 200 µM, respectively). BP7033Br blocked 10% of MDA-MB-231 cells into the G0/G1 phase (*P* = 0.0002) and diminished the cell number in the S phase (*P* = 0.017, Fig. 3C). In contrast, BP7033Br ALK increased the number of MDA-MB-231 cells into the S phase (*P* = 0.005) accompanied by a reduction of the proportion of cells in G0/G1 phase (*P*<0.001). Concerning the D3H2LN cells, BP7033Br increased about 20% the number of cells (*P* = 0.048, Fig. 3D) in the G0/G1 phase, as observed for MDA-MB-231 cells, and completely inhibited the proportion of cells in the G2/M phase. Also, BP7033Br ALK increased the number of D3H2LN cells into the S phase to about 15% (*P* = 0.006, Fig. 3D).

### BP7033Br and BP7033Br ALK induced MDA-MB-231 breast cancer cell death

**Figure 4:**
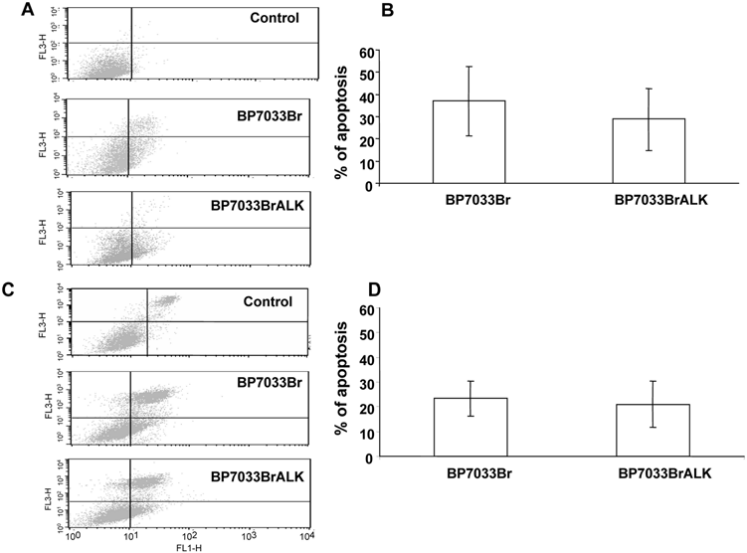
BP7033Br and BP7033Br ALK induced MDA-MB-231 breast cancer cell apoptosis. Preconfluent MDA-MB-231 (A) and D3H2LN (C) cells were treated with 500 µM BP7033Br or 200 µM BP7033Br ALK for 72 h in a serum-containing medium. Percentages of MDA-MB-231 (B) and D3H2LN (D) were evaluated as described in “Materials and Methods”. Each column represents a mean (±SD) of three independent experiments.

The Ann-V-positive/PI-negative population corresponds to cells in an early apoptotic phase and the Ann-V-positive/PI-positive one to cells in a late apoptosis phase (Fig. 4). We evaluated the apoptotic effect of efficient doses of both BPs on breast cancer cell lines. Both BP7033Br (500 µM) and BP7033Br ALK (200 µM) induced apoptosis of the MDA-MB-231 cells inducing the same amount of cells in both early and late apoptosis (Fig. 4A). Total percentage of MDA-MB-231 cells in apoptosis (late plus early) induced by BP7033Br and BP7033BR ALK is about 37 and 25% as compared to control, respectively (Fig. 4B). The percentage of apoptotic cells was not significantly different between the two BPs treatments(*P* = 0.069) although concentration used for BP7033Br ALK was lower (200 µM) Concerning D3H2LN cells, the effect of the two BPs was slightly less effective (20%) as compared to the MDA-MB-231 parental cells (Fig. 4C and 4D).

### BP7033Br and BP7033Br ALK inhibited MDA-MB-231 cells migration, invasion and MMP-9 and MMP-2 proteolytic activation

**Figure 5:**
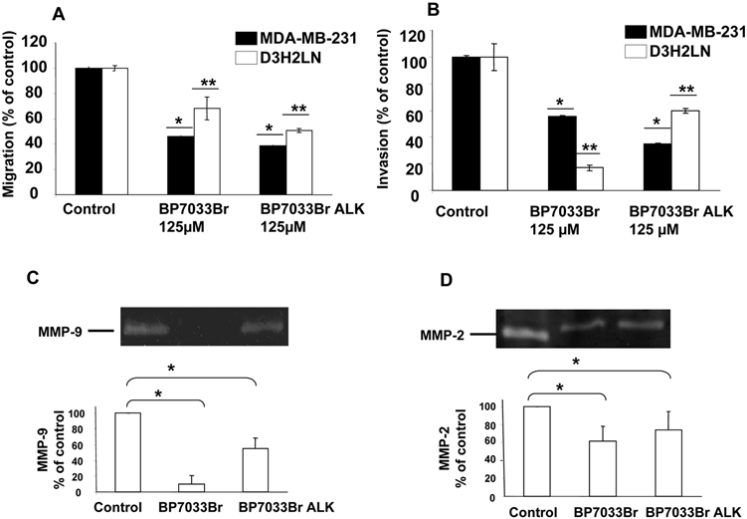
BP7033Br and BP7033Br ALK inhibited MDA-MB-231 breast cancer cell migration, invasion, MMP-9 and MMP-2 activities. BPs inhibited MDA-MB-231 breast cancer cell migration (A) and invasion (B). Cells (2.5×10^5^) with 125 µM of BPs were added to each 8 µm-insert in the upper chamber of boyden chamber. After 24 h, cells invading the chamber were fixed, stained and counted as described in “Materials and Methods”. BPs inhibited MMP-9 and MMP-2 activities (C and D, respectively). Lyophilized conditioned media were normalized to the number of cells and subjected to 10% SDS-polyacrylamide gels containing 1 mg/mL gelatine. Lane 1, 2 and 3 represent the control, BP7033Br and BP7033Br ALK conditioned medium of treated cells, respectively. Each column represents a mean (±SD) of three independent experiments. *P_versus MDA-MB-231 control_<0.05, ** *P*_versus D3H2LN control_<0.05.

We next explored the effect of BPs on the migration of the two MDA-MB-231 cell lines (Fig. 5A). MDA-MB-231 cells as well as D3H2LN subpopulation ones, migrated through the lower chamber side when 10% FCS-DMEM was used as chemo attractant. BP7033Br (125 µM) inhibited about 55% the migration of MDA-MB-231 cells but was less efficient on the D3H2LN cell migration (32%, *P*<0.001). The same concentration of BP7033Br ALK induced stronger motility inhibition of both cell lines (62 and 50%, respectively). Since invasion is also important in the metastasis process, we investigated the inhibition induced by BPs on the MDA-MB-231 cell invasion (Fig. 5B). In the presence of 10% FCS-DMEM, MDA-MB-231 cell lines invaded the inserts coated with matrigel. In contrast to the results obtained with MDA-MB-231 cell migration, we observed a better inhibition of D3H2LN invasion by BP7033Br as compared to BP7033Br ALK (84% versus 40%, *P*<0.001). Concerning the MDA-MB-231 cells, BP7033Br ALK inhibited cell invasion about 65% whereas BP7033Br reduced it about 45%.

To further study the BPs effects on invasion, we evaluated the effect of BPs on MMP activity in the D3H2LN cells (Fig. 5). D3H2LN cells showed activation of MMP-9 and MMP-2 (lane 1, Fig. 5C and 5D). BP7033Br strongly inhibited the activation of MMP-9 (Fig. 5C, lane 2, *P* = 0.0037). In contrast, BP7033Br ALK only reduced about 45% the MMP-9 activity (Fig. 5C, lane 3, *P* = 0.0065). In addition, BP7033Br inhibited about 40% (*P* = 0.001) MMP-2 activity whereas BP7033Br ALK reduced MMP-2 activation about 25% (*P* = 0.01, Fig. 5D, Lane 2 and 3, respectively).

### Both BP7033Br and BP7033Br ALK inhibited the D3H2LN tumor growth and angiogenesis in nude mice

**Figure 6:**
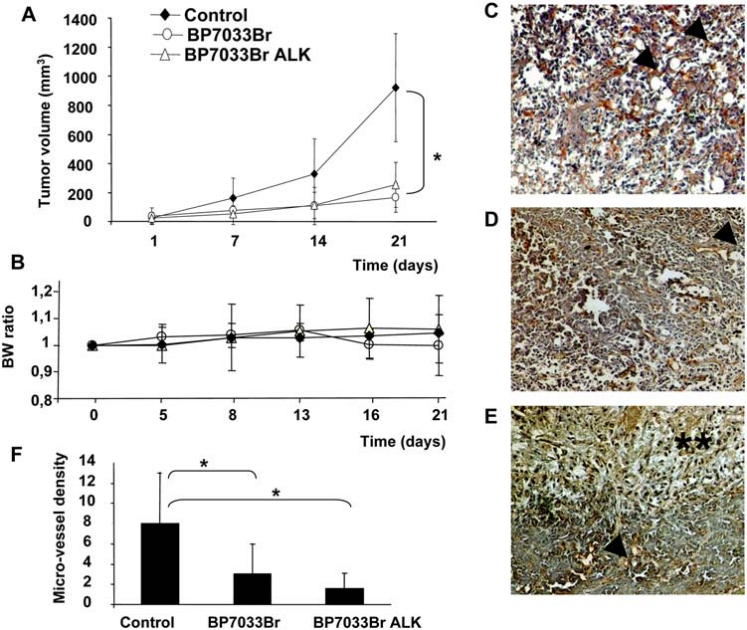
BP7033Br and BP7033Br ALK inhibited D3H2LN tumor growth and esterified analogue completely inhibited angiogenesis. (A) D3H2LN cells were inoculated in nude mice as described in “Materials and Methods”. After 1 week, mice were treated with BPs (11 mg/kg), twice a week, for 21 days. Each column represents the mean of tumor volume (mm^3^) (±SD, n = 7). Body weight (BW) ratio was determined for each group (B). Endothelial cells in tumor sections were stained in controls (C), BP7033Br (D) and BP7033Br ALK (E) Microvessels were indicated by arrows and necrosis area by double asterisks (magnification ×100). Quantification of micro-vessel density (F). Each column represents a mean (±SD) of three independent experiments. *P_BP7033Br and BP7033Br ALKversus control_<0.05.

All 7 mice developed tumors within one week after D3H2LN inoculation and the BPs treatments were initiated at the end of the first week (Fig. 6A). Both BP7033Br and BP7033BR ALK used at 11 mg/kg (286 µg/mouse) twice a week significantly inhibited D3H2LN growth after 21 days as compared to control (*P* = 0.034 and *P* = 0.038, respectively). D3H2LN tumor growth was inhibited by about 80% with both BPs. All mice treated with the two BPs were alive at the end of the treatment and did not present significant loss of body weight (Figure 6B). In addition, no macroscopic differences were observed between treated and control mice liver and kidney after autopsy (data not shown).

GSL-1 selectively labelled the endothelial cells and thus enabled to determine the relative density of micro-vessels in D3H2LN tumors xenografted in nude mice (Fig. 6C–E). BP7033Br ALK treated tumors exhibited large areas of necrosis (Fig. 6E). BP7033Br ALK treatment strongly reduced the micro-vessel density (80%) in viable field of tumors as compared to control and BP7033Br treatments (Fig. 6F, *P* = 0.01 and *P* = 0.047, respectively).

### Only BP7033Br ALK inhibited D3H2LN metastasis

**Figure 7:**
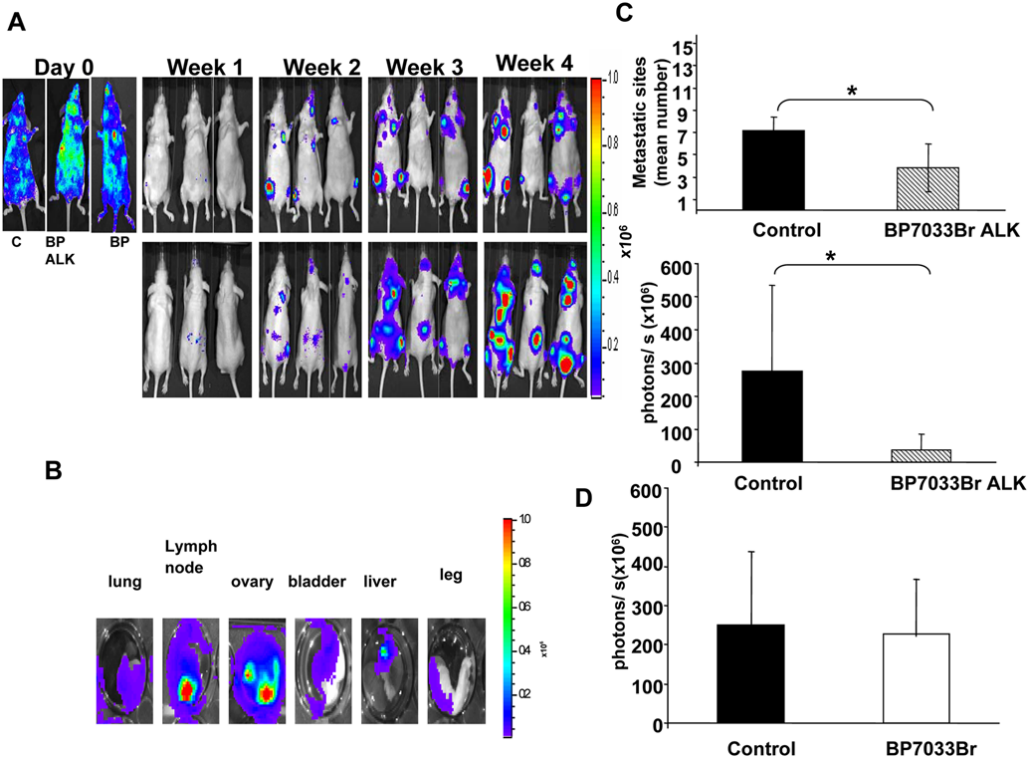
Only BP7033Br ALK inhibited D3H2LN metastasis. D3H2LN cells were injected into the left ventricle of nude mice (n = 7). Day 0 showed the successful intracardiac cells injection. Within 2 weeks, when metastasis were initiated, mice were treated with BP7033Br ALK or BP7033Br (A). At the indicated days, the bioluminescence images were acquired for control (c, left panel) and BPs treated mice (BP7033BrALK and BP7033Br middle and right panel, respectively). *Ex vivo* data confirm soft tissue metastasis from D3H2LN cells injection (B). Quantification of the mean metastatic sites and the photons/s after BP7033Br ALK treatment (C). Quantification the photons/s after BP7033Br treatment (D). Each column represents a mean (±SD) of three independent experiments. *P_versus control_<0.05.

Since D3H2LN was described to develop bone and soft tissues metastasis after intracardiac injection in nude mice [15], we further studied the effect of both BPs on this D3H2LN metastasis model (Fig. 7). Animals (n = 7) with successful intracardiac injection at day 0 (Fig. 7A) were treated with each BPs at 11 mg/kg twice a week during 4 weeks. The control group received sterile PBS. Injections started the first week when micrometastasis appeared. Within 2 weeks after intracardiac injection, all animals exhibited metastasis at multiple sites in the head, thorax, abdomen, legs or spine (Fig. 7A). *Ex vivo* imaging of the different tissues after the final imaging *in vivo*, showed bone lesions (legs) as well as lung, lymph nodes, ovary, bladder and liver ones (Fig 7B). Treatment with BP7033Br ALK at 11 mg/kg twice a week, induced inhibition about 80% of luminescence signalling and 50% reduction of the mean metastatic sites number per animal (*P* = 0.01 and 0.08, respectively, Fig. 7C). In contrast, BP7033 did not show any significant reduction in metastasis bioluminescence signals or number sites (Fig. 7D).

## Discussion

BPs represented an emerging class of drugs for cancer therapy. In this work, we demonstrated the efficacy of a new class of non-N-BPs which exhibited higher antiproliferative activities on breast cancer cells compared to previously described non-N-BPs such as clodronate [16]. Both types of m-bromobenzyl BPs inhibited the viability of several breast cancer cell lines with different estrogen-receptor statuses. We showed that the esterified BP was the more effective on estrogen-responsive cells since the maximal inhibition was reached at 250 µM in contrast to non esterified BP that did not induce maximal inhibition even at 1 mM. In addition, at 250 µM, BP7033Br ALK is effective on cells independently from the estrogen-receptor status. Both types of our BP inhibited viability of estrogen non-responsive cells and particularly that of MDA-MB-231 and D3H2LN cells, the last cell line being the more aggressive ones. It was worth to note that we demonstrated for the first time a dramatic improvement of antiproliferative effect of non-N-BPs on breast cancer cells since clodronate at the same concentration range (200 µM) and the same time-treatment (72 h) did not reduce MDA-MB-231 cell viability [17]–[19]. In addition, clodronate demonstrated mitogenic effects via MCF7 estrogenic receptor [16] and we never observed this effect with our BPs. Based on our results, it appears also that BP7033Br ALK antiproliferative effect is estrogen-receptor-independent. The occurrence of this new effect of non-N-BPs could result from the addition of aromatic functions in the side chain. Heterocycle in the side chain was implicated in the induction of cell apoptosis by preventing the prenylation of signalling proteins such as Ras or Rho [20]. We previously demonstrated the inhibition of Ras processing using non bromo-containing BP7033 [21]. Also, the addition of phenyl function in the side chain of BPs rendered the catalytic pocket of geranyl and/or farnesyl synthase enzymes of the mevalonate pathway more accessible [14], [22]. BP7033Br ALK reduced MDA-MB-231 and D3H2LN cell viabilities about 90% with a concentration 4-fold inferior to that of BP7033Br. These data were in agreement with our previous results on epidermoid A431 cell proliferation that showed a beneficial effect of esterification of the phosphonic groups [12], [13]. Our hypothesis was that such esterified compounds rendered BPs more hydrophobic increasing their cell uptake and could therefore act like prodrugs releasing active BP into the cells. In accordance, characterisation of the hydrophilicity demonstrated a shift toward lipophilicity of the BP7033Br ALK compound (Log P values of −0.75 versus −031, respectively). Alternatively, one could also hypothesize that esterified BPs had their own mechanisms since they blocked the cells into the S phase while non esterified BPs inhibited the G0/G1 cell phase transition. On the other hand, both type of BPs (esterified or not) induced cell death apoptosis of both MDA-MB-231 and D3H2LN cells. These results are interesting since D3H2LN had a metastatic potential greater than MDA-MB-231 and consequently could be more resistant to apoptosis as it was described for metastatic cells [23], [24]. Both BPs induced strong D3H2LN metastatic cell apoptosis but the concentration of the esterified analogue used was 2-fold lower. Also, the two BPs inhibited migration of MDA-MB-231 cell lines with a more important effect obtained with BP7033Br ALK. In contrast, BP7033Br ALK was less effective in inhibiting D3H2LN cell lines invasion concomitant with a less important effect on MMP-9 and MMP-2 activities. BP7033Br strongly inhibited MMP-9 and MMP-2, the major form of metalloproteinases present in extracellular matrix. Since MMPs are zinc-dependent endopeptidases, we speculated that the reduction of BP7033Br ALK effect could be due to a reduction of available phosphonic acid groups able to chelate zinc and consequently inhibit MMPs. These results were in agreement with previous studies which showed that MMPs inhibition activity by BPs was related to zinc chelation [2]. However, we hypothesised that release of BP7033Br ALK with free phosphonic group could be more important in *in vivo* system because of the presence of phosphodiesterases in serum. Also, we found that these BPs had no influence on MMPs expression (data not shown).

Interesting results were the BPs antitumor effects observed on D3H2LN xenografts growth and metastasis. D3H2LN cells were obtained from a MDA-MB-231 subclone isolated from a lymph node metastases and induced an increased xenograft tumor growth as compared to parental cells when injected *in vivo* [15]. Both BP7033Br and BP7033Br ALK inhibited about 80% the D3H2LN tumor growth after intratumoral injection of about 286 µg BPs per mouse twice a week during 21 days. We established that this new class of BPs was the more potent among the current non N-BPs since clodronate, even used at 1600 mg twice daily during several weeks (as compared to BP7033 ALK corresponding human dose of 770 mg twice a week during only 2 weeks) failed to reduce primary tumor growth [25]. Also, we found that our BPs were 10-fold more potent than the non halogenated phenyl analogues [26]. In addition, BP7033Br ALK better inhibited D3H2LN vessel density than BP7033Br. This point is also supported by the large necrosis area not detected in BP7033Br treated tumors. In addition, we can not exclude that these large necrosis areas could also be due to a greater amount of esterified BP penetrating into tumor to induce cell death. As compared to N-BPs, it was noteworthy that risedronate or ibandronate failed to inhibit MDA-MB-231 tumor growth in nude mice [27], [28]. Furthermore, no pre-clinical works demonstrated an antiproliferative effect of zoledronate on primary breast tumor growth in nude mice. The only study demonstrating an inhibition effect of zoledronate on primary tumor growth used mesothelioma tumors which involved calcification that could uptake the drug [29]. In addition, the efficacy of zoledronate on bone metastasis seems to be supported by its affinity for osseous tissues rather than its direct antiproliferative effect on tumor cells [25]. Also, zoledronate was a compound rapidly eliminated from plasma, resulting in renal excretion, rapid bone or calcified tissues uptake and accumulation partly due to its phosphonic functions. Here, we showed that the symmetrical esterification of the phosphonic groups may improved BPs soft tissue bioavaibility limiting osseous or calcified tissue uptake. Also, as their chemical structures are close to the apomine BP which presented aliphatic ester group, we suggested that their half-life will be close to that found for this drug (156 h with micromolar plasma concentration, [30]). Thus, esterified BP7033Br ALK abrogated angiogenesis, both soft tissue and bone metastasis whereas BP7033Br did not. Noteworthy, in BP7033Br treated mice, luminescence signalling of leg osseous metastasis was not significantly reduced because 2/7 mice did not respond to the treatment in contrast to BP7033Br ALK treatment that induced significant reduction (data not shown). Indeed, the esterified functions seem to be important for the BPs distribution within the systemic system and less for local injection (subcutaneous tumors) since the two N-BPs studied both inhibited D3H2LN xenograft growth. To note, was the toxic adverse effects of N-BPs inducing osteonecrosis of treated patients [7] and no apparent side effect reported with non N-BPs in the present study.

In conclusion, esterified m-bromobenzyl non N-BPs constituted a new class of drugs with potent direct antitumor, antiangiogenic and antimetastatic efficacy on breast tumors.

## Materials and Methods

### Bisphosphonates synthesis and hydrophilicity characterisation

The bisphosphonate molecule evaluated in this study corresponds to [2-(3-Bromo-phenyl)-1-hydroxy-1-phosphono-ethyl]-phosphonic acid (MW: 400 g/L, Fig. 1). This compound (BP7033Br) was synthesised as previously described [31], [32]. The novel esterified analogue, {2-(3-Bromo-phenyl)-1-hydroxy-1-[hydroxy-(4-methoxy-phenoxy)-phosphoryl]-ethyl}-phosphonic acid mono-(4-methoxy-phenyl) ester named BP7033Br ALK (MW: 572 g/L, Fig. 1), was synthesised following the same methodology in two steps. Briefly, a very reactive species, the bis(trimethylsilyl)(*p*-methoxyphényl) phosphite, was reacted following an Arbusov (1) reaction with an activated carboxylic function and then the intermediate was hydrolysed (2). Log *P* values were determined using a shake-flask method as described previously [33]. Aqueous sodium chloride (0.9% w/v) and n-octanol phase were saturated for a week. Bisphosphonates were dissolved at a concentration of 0.02 M in the aqueous phase (2.5 mL). An equal volume of saturated n-octanol was added and the solution was mixed. The content of aqueous phases was determined by UV spectroscopy respectively at 268 and 278 nm (BP7033 Br and BP7033Br ALK). The logarithm of the ratio of BPs concentrations in the organic and aqueous phases was calculated to determine following log *P* values: log *P*_(BP7033Br)_ = −0.75 ; log *P*_(BP7033BrALK)_ = −0.31. In addition, 0at physiological pH both compounds BP7033 Br and BP7033Br ALK were readily soluble at 2 mM concentration in both distilled water and aqueous sodium chloride solution (0.9% w/v). BP7033Br ALK becomes insoluble in water at concentration higher than 20 mM and below 10 mM in aqueous sodium chloride solution. BP7033 Br was still soluble at 50 mM in both solutions.

### Cell lines

The human breast adenocarcinoma MDA-MB-231, T47D, MCF-7 cell lines were obtained from the American Type Culture Collection (Manassas, VA, USA). SKBR3 were kindly obtained from Dr Cavailles (U824, CRLC, Montpellier, France). Cells were maintained in Dulbecco's minimal essential medium supplemented with 10% fetal bovine serum (FBS), 1% sodium pyruvate and antibiotics (1% penicillin sodium and 1% streptomycin) at 37°C in a humidified atmosphere containing 5% carbon dioxide. D3H2LN cell line isolated from MDA-MB-231 lymph node metastasis was obtained from Caliper Life Sciences (Alameda, CA, USA). D3H2LN cell line was a subclone selected from a MDA-MB-231 stable clone expressing firely luciferase. MDA-MB-231 cells expressing luciferase were injected into the mammary fad pad of nude mice and after 12 weeks of growth *in vivo*, they were harvested and re-propagated *in vitro*. This subclone was injected once more into the mammary fad pad of nude mice to yield a second cell line D3H2LN, harvested from a lymph node metastasis [15]. D2H2LN cells were cultured in Minimum Essential Medium with Earl's Balanced Salts Solution MEM/EBSS medium supplemented with 10% fetal bovine serum, 1% nonessential amino acids, 1% L-glutamine, and 1% sodium pyruvate and antibiotics (all from Hyclone, Logan, UT, USA) at 37°C in a humidified atmosphere containing 5% carbon dioxide.

### Cell viability

MDA-MB-231 and D3H2LN cell viability was assessed using the MTT-microculture tetrazolium assay [34]. The cells were then incubated with different concentrations of BP7033Br or BP7033Br ALK, for 72 h at 37°C in a 5% CO_2_-incubator. Optical density was measured at 570 nm using a Labsystems Multiskan MS microplate reader.

### Cell Cycle Analysis

Cells (2×10^5^) were incubated with 500 µM BP7033Br or 200 µM BP7033Br ALK for 72 h. Adherent cells were harvested, washed with cold PBS, then fixed with ice-cold 70% ethanol at −20°C for 1 h. Cells were then treated with RNAse A (200 µg/mL) and stained with propidium iodide (50 mg/mL) at room temperature for 30 min in the dark. After incubation, the red fluorescent cells were analysed by flowcytometer (Becton Dickinson). DNA histograms were created using Cell Quest software (Becton Dickinson) analysing 1×10^4^ events per sample. The relative distribution of cells in the phases of the cell cycle was calculated with ModFiLT software (Becton Dickinson).

### Cell death detection

To reveal a phosphatidylserine translocation specific to early apoptosis stage, the cells were stained with a FITC-labelled annexin-V (Ann-V). The ultimate stage of apoptosis or the first stage of necrosis was revealed by incorporation of PI, which enters into the cells when cell membrane damage has occurred. Cells (1×10^5^) were incubated with 500 µM BP7033Br or 200 µM BP7033Br ALK for 72 h in serum-containing medium. No organic vehicle was used since both BPs were soluble in water or culture medium until 20 mM. In addition, cells were harvested and apoptotic cells were determined using the Annexin V-FITC Apoptosis Detection kit (Beckman coulter, Fullerton CA, USA). Then, the cells were analysed by flow cytometry (Becton Dickinson, Heilderberg, Germany).

### Cell migration and invasion assay

Cell migration experiments were performed using migration chambers containing 8 µm pore size (Becton Dickinson). Cells (2,5×10^5^) with 125 µM of BPs were added to each insert (upper chamber). 10% FCS-DMEM was used as chemoattractant. For invasion assays, Boyden invasion chambers with 8 µm pore size filters coated with Matrigel (Falcon, MA, USA) were used. After 24 h incubation, non-migrated cells in the upper chambers were removed by wiping cells with a cotton swab. Then, cells on the lower filter face were fixed, stained and counted. Results were expressed as a percentage, relative to controls normalised to 100%. Experiments were performed in triplicate.

### Gelatine zymography

The serum-free conditioned media of MDA-MB-231 and D3H2LN cells treated with both BPs for 24 h in 6-wells plates were lyophilized. Lyophilized conditioned media were normalized to the number of cells mixed with non-reducing LaemmLi buffer and subjected into 10% SDS-polyacrylamide gels containing 1 mg/mL gelatine in the presence or absence of BP. The gel was washed 3 times at room temperature in a solution containing 2.5% (v/v) Triton X-100 and incubated at 37°C for 24 h in 50 mM Tris/HCL, pH 7.5, 0.2 M NaCl, 5 mM CaCl2 and 0.05% Brij 35. The gel was stained for 60 min with 0.25% (w/v) R-250 Coomassie blue in 45% (v/v) methanol/10% (v/v) acetic acid and destained in 25% methanol (v/v)/10% acetic acid (v/v).

### Xenografts in nude mice

All *in vivo* experiments were carried out with local ethical committee approval and accordingly to the UKCCCR guidelines. Animals were kept in a temperature-controlled room on a 12: 12 light-dark schedule with food and water *ad libitum*. D3H2LN cells (2×10^6^ cells) were inoculated subcutaneously (s.c) in 4-week-old athymic nude mice (nu/nu, Janvier, France, n = 21). Then, mice were arbitrarily placed in control (n = 7) and in each BPs treated group (n = 7). The administration of BP7033Br or BP7033Br ALK (11 mg/kg) started 1 week after cell inoculation when tumors reached 30 mm^3^. The concentration of 11 mg/kg corresponds to about 250 µM for BP7033 ALK and 500 µM for BP7033 if considering the MW of both BPs and the mean mouse weight of about 30 g. Control group received 0.1 mL of PBS 1×. Treatments or PBS solutions administration were intratumoral twice a week for 4 weeks. Tumor volume was measured once a week along to major axes using callipers. Tumor volume (mm^3^) was calculated as following: V = 4/3πR_1_^2^R_2_ where R_1_<R_2_

Tolerability of the dose used (11 mg/kg) was evaluated according to the same protocol used for BPs injection on each tumor and tumor-free healthy mice (n = 5). Each animal was weighted once a week during the treatment.

### Immunohistochemical analysis

Tumour specimens were fixed in 4% paraformaldehyde and embedded in to paraffin using standard procedure. Routinely, 5 µm-sections were stained in haematoxylin and eosin. For immunohistochemical studies the sections were deparaffinized and rehydrated. Endogenous peroxidase was inactivated with 3% H_2_O_2_ and washed in TBS (Tris 0.05 M, NaCl 1.5 M, pH 7.6) followed by pre-incubation in 10% normal goat serum for 1 h at room temperature. Endothelial cells were specifically labelled with GSL-1 isolectin B4 (Vector Laboratories, Burlingame, CA, U.S.A.). The sections were incubated for 1 h with the 1∶50 diluted GSL-1 isolectin at room temperature. The sections were then incubated with goat antibody against GSL-1 isolectin B4 (1∶400 dilution, Vector Laboratories) for 30 min, washed with TBS and incubated with biotinylated rabbit anti-goat immunoglobulins (1∶400 dilution; Dako,Glostrup, Denmark) for 20 min in a moist chamber at room temperature. After three washes with TBS, the samples were incubated with streptavidin-biotin peroxidase (LSAB kit;Dako) for 10 min using diaminobenzidine tetrahydrochlorideas the chromogen. Finally, the slides were washed in water and counterstained with hematoxylin. Intratumor microvessel areas were determined as described previously [20]. For each tumour, 10 randomly selected non serial sections were studied.

### Intracardiac experimental metastasis model

Female nude mice (8–10 weeks) were anasthetized by intraperitoneal injection of 120 mg/kg ketamine and 6 mg/kg xylazine on the day of injection and by exposure to 1–3% isoflurane on subsequent imaging days. On day 0, anaesthetized animals were injected with D3H2LN (1×10^5^cells) in 100 µl sterile PBS into the left ventricle of the heart by non surgical means. Anesthetized mice were placed in the IVIS™ Imaging System (Xenogen) and imaged from both dorsal and ventral views approximately 5 min after intraperitoneal injection of D-luciferin (Caliper Life Science). A successful intracardiac injection was indicated on day 0 by systemic bioluminescence distributed throughout the animal. Only mice with satisfactory injection continued the experiment and were treated by intraperitoneal injection of PBS (control) or with both BPs (11 mg/kg). Assessment of subsequent metastasis was monitored *in vivo* once a week by imaging for up to 3 weeks.

### Bioluminescent Imaging

Bioluminescence images were acquired with the IVIS imaging system (Xenogen) at 5 min after intraperitoneal injection of D-luciferin to anesthetized animals as described above. Acquisition times of the beginning of the time course started at 5 min and were reduced in accordance with signal strength to avoid saturation. Analysis was performed using LivingImage software (Xenogen) by measurement of photon flux (photon/s/cm^2^) with a region of interest (ROI) drawn around the bioluminescence signal.

### Statistical analysis

Statistical significance was determined by the Student's t-test. P<0.05 was considered significant
